# Natural Compound Mixture, Containing Emodin, Genipin, Chlorogenic Acid, Cimigenoside, and Ginsenoside Rb1, Ameliorates Psoriasis-Like Skin Lesions by Suppressing Inflammation and Proliferation in Keratinocytes

**DOI:** 10.1155/2020/9416962

**Published:** 2020-10-22

**Authors:** Uy Thai Nguyen, Ly Thi Huong Nguyen, Bo-Ae Kim, Min-Jin Choi, In-Jun Yang, Heung-Mook Shin

**Affiliations:** ^1^Department of Physiology, College of Korean Medicine Dongguk University, Gyeongju 38066, Republic of Korea; ^2^Division of Biomedicinal & Cosmetics, College of Sciences & Technology, Mokwon University, Daejeon 302-729, Republic of Korea

## Abstract

Herbal combinations of Rhei Radix et Rhizoma, Gardeniae Fructus, Cimicifugae Rhizoma, and Ginseng Radix have been used in traditional formulas to treat the symptoms of heat and dryness. This study investigated the therapeutic effects of a natural compound mixture (PSM) of these herbal combinations, containing emodin, genipin, chlorogenic acid, cimigenoside, and ginsenoside Rb1, for the treatment of psoriasis and its underlying molecular mechanisms. PSM was applied topically to the dorsal skin lesions of imiquimod- (IMQ-) induced C57BL/6 mice, and the expression of the proinflammatory mediators was investigated. The topical application of 1% PSM reduced psoriasis-like symptoms in IMQ-induced C57BL/6 mice significantly. PSM also attenuated the production of IFN-*γ*, IL-1*β*, and IL-6 in skin lesions. Histological analysis showed that PSM had antipsoriatic effects by reducing the lesional epidermal thickness. Either M5 (IL-1*α*, IL-17A, IL-22, oncostatin M, and TNF-*α*, 10 ng/ml each) or IL-22- (100 ng/ml) stimulated HaCaT cells were used to examine the efficacy and underlying mechanism of PSM. In M5-stimulated HaCaT cells, PSM inhibited the production of C-X-C motif chemokine ligand (CXCL) 10 and C-C motif chemokine ligand (CCL) 20 effectively. Moreover, compared to the use of a single compound, it had synergistic inhibitory effects in CXCL8 production. PSM suppressed the phosphorylation of ERK1/2, p38, and STAT3 signaling pathways in M5-stimulated HaCaT cells. Furthermore, PSM reduced the proliferation rate and K16 and K17 expressions in IL-22-stimulated HaCaT cells by inhibiting the Akt/mTOR signaling pathway. These results suggest that PSM may have a therapeutic potential in the treatment of psoriasis lesions.

## 1. Introduction

Psoriasis is an inflammatory skin disorder that affects approximately 2% of the world's population. The hallmarks of psoriasis are chronic inflammation, excessive proliferation, and aberrant differentiation in keratinocytes, leading to erythematous and scaly plaque on the skin [1]. Although the pathogenesis of psoriasis is not completely understood, recent studies have demonstrated an important role of the T helper 17 (Th17) cells in the development of the disease [1, 2]. Th17 cells can produce several cytokines, such as IL-17A, IL-17F, and IL-22, which activate various inflammatory cascades and keratinocyte proliferation in skin lesions [2]. Moreover, activated keratinocytes can attract immune cells to skin lesions by producing several chemokines, such as C-X-C motif chemokine ligand 8 (CXCL8), CXCL10, and C-C motif chemokine ligand 20 (CCL20). The release of these chemokines can exacerbate inflammation in psoriatic lesions through the recruitment of neutrophil, Th1, and Th17 cells [3–5].

There are many therapeutic options available for treating psoriasis. Topical glucocorticoids are indicated as the first and most effective drugs for the treatment of psoriasis, but the long-term application of glucocorticoids might lead to numerous side effects, including skin atrophy, impairment of skin barrier function, and wound healing [6]. Phototherapy such as exposing the skin to natural sunlight or narrow-band ultraviolet B (NB-UVB) was widely prescribed by a dermatologist to treat psoriasis [7]. A common approach in alternative therapy is the use of natural products, including medicinal herbs and extracted compounds [8, 9]. In traditional medicine, medicinal herbs are often prescribed in combination. This therapeutic strategy using an herbal mixture can act on multitargets by exhibiting synergistic effects or reducing the adverse effects. Multiherbal formulas are a combination of multiple compounds used to cure the disease [10]. Such a combination of natural compounds can solve the treatment disadvantage of a single compound and have synergistic effects [11, 12]. A previous study found that a combination of compounds can modulate the complex and diverse pathological factors of psoriasis by acting on multitargets [13].

**Figure 1:**
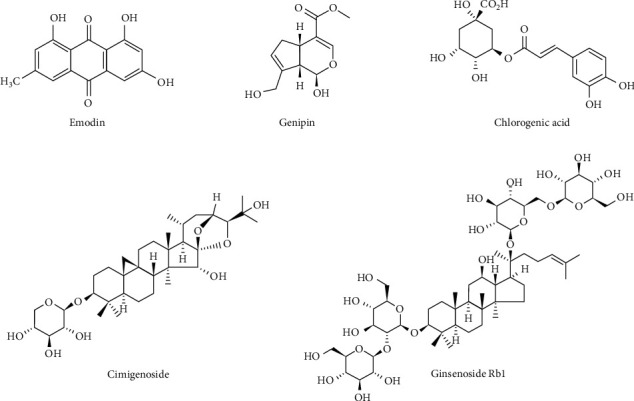
Chemical structures of emodin, genipin, chlorogenic acid, cimigenoside, and ginsenoside Rb1.

In traditional medicine, heat and dryness are the two main Zheng of psoriatic patients [14]. Herbal combinations of Rhei Radix et Rhizoma, Gardeniae Fructus, Cimicifugae Rhizoma, and Ginseng Radix are used in a traditional formula called Rhubarb Pill to treat the symptoms of heat and dryness. These combinations are also used to treat swollen eyelids in a formula called antelope horn powder in Dongui Bogam, compiled by HeoJun (1539–1615). Hence, a combination of five natural compounds (PSM) was prepared: emodin, genipin and chlorogenic acid, cimigenoside, and ginsenoside Rb1 (Figure 1), which are the active ingredients of Rhei Radix et Rhizoma (the rhizome of *Rheum palmatum* Linne), Gardeniae Fructus (the ripe fruit of *Gardenia jasminoides* Elli), Cimicifugae Rhizoma (the rhizome of *Cimicifuga heracleifolia* Komarov), and Ginseng Radix (the root of *Panax japonicus* C. A. Meyer), respectively. In the present study, in vivo and in vitro experiments were conducted to evaluate the effects of this mixture on psoriasis-like symptoms, including inflammation and hyperproliferation.

## 2. Materials and Methods

### 2.1. Chemicals and Reagents

Emodin, genipin, chlorogenic acid, cimigenoside, and ginsenoside Rb1 were purchased from ChemFaces (Hubei, China). Imiquimod (IMQ) was obtained from 3M Health Care (Leicestershire, England). High glucose Dulbecco's modified Eagle's medium (DMEM) was acquired from WelGENE Inc. (Gyeongsangbuk, Korea). Fetal bovine serum (FBS) and antibiotics were supplied by Invitrogen Inc. (Carlsbad, CA, USA). U0126, SB202190, SP600125, cryptotanshinone, and wortmannin were obtained from Sigma-Aldrich (St. Louis, MO, USA). Human CXCL8, CXCL10, and mouse IFN-*γ*, IL-1*β*, IL-6 ELISA kits were purchased from KOMA Biotech Inc. (Seoul, Korea). The human CCL20 ELISA kit was bought from R&D system (Minneapolis, MN, USA). The antibodies for phosphorylated extracellular signal-regulated kinase (p-ERK), phosphorylated c-Jun N-terminal kinase (p-JNK), phosphorylated p38 (p-p38), phosphorylated signal transducer and activator of transcription 3 (p-STAT3), phosphorylated Akt (p-Akt), phosphorylated mammalian target of rapamycin (p-mTOR), ERK, p38, JNK, STAT3, Akt, and mTOR were obtained from Cell Signaling Technology (Danvers, MA, USA). The antibodies against keratin 16 (K16) and K17 were procured from Abcam (Cambridge, MA, USA). HRP-conjugated anti-*β*-actin was supplied by Sigma-Aldrich (St. Louis, MO, USA).

### 2.2. Animals and Treatment

C57BL/6 (8-week-old male) mice were purchased from Laboratory Animal Resource Center (Seoul, Korea). The Institutional Animal Care and Use Committee of Dongguk University approved all the animal experimental procedures (approval no. IACUC-2017-015). The mice were divided randomly into five groups (*n* = 7): control group (NC), imiquimod (5%) cream group (the IMQ group), 0.1% psoriasis mixture group (the 0.1% PSM group), 1% psoriasis mixture group (the 1% PSM group), and 0.1% dexamethasone group (the DEX group). After the backs of the mice were shaved, 62.5 mg of 5% IMQ cream was applied daily to induce psoriasis-like skin lesions for six consecutive days on the mice in the IMQ, 0.1% PSM, 1% PSM, and DEX groups. PSM was prepared by dissolving equal amounts of each compound in a 3 : 1 mixture of acetone and olive oil, followed by sonication for 10 min. Previous research has shown that the acetone:olive oil vehicle did not induce epidermal hyperplasia or increase the proinflammatory cytokines level [15]. “PSM 1.0%” means a composition containing 2 mg/ml of emodin, genipin, chlorogenic acid, cimigenoside, and ginsenoside Rb1. The dorsal skin was pretreated with the 0.1% PSM, 1% PSM, or DEX 1 h prior to IMQ application.

After six days of treatment, the mice were sacrificed, and skin tissues were collected. The psoriasis severity was assessed at the end of the experiment using the Psoriasis Area Severity Index (PASI) with measurements of skin redness, scaling, and thickness. The scores were interpreted as follows: 0, no symptoms; 1, mild; 2, moderate; 3, severe; and 4, very severe. The body weight and spleen weight of each mouse were recorded. The skin lesion of their backs was excised and stored in 4% paraformaldehyde for histological analysis, or they were homogenized using a tissue extraction reagent for protein expression analysis.

### 2.3. Histological Analysis

The dorsal skin sample from each mouse was fixed in 4% paraformaldehyde and embedded in paraffin. The sections were cut at 5 *μ*m and stained with hematoxylin and eosin (H&E). Histological analysis was performed using a Lionheart FX Automated Microscope and Gen5 Imager software (BioTek Instruments Inc., Winooski, VT, USA).

### 2.4. Cell Culture and Treatments

HaCaT cells (a human keratinocyte cell line) were cultured in DMEM supplemented with 10% FBS and 1% penicillin-streptomycin at 37°C in a 5% CO_2_-humidified environment. The medium was changed every two days during incubation, and the cells were made quiescent by starvation in a serum-free medium for 24 h. The cells were pretreated with emodin, genipin, chlorogenic, cimigenoside, ginsenoside Rb1 (10 *μ*M), PSM (10 or 50 *μ*M), or DEX (10 *μ*M) for 1 h before stimulation with M5 (IL-1*α*, IL-17A, IL-22, oncostatin M, and TNF-*α*, 10 ng/ml each) or IL-22 (100 ng/ml) for the indicated time. PSM was prepared by mixing five compounds with the same concentration (*μ*M) in DMSO, followed by sonication for 10 min. For example, “PSM 50 *μ*M” means a composition containing 10 *μ*M of emodin, genipin, chlorogenic acid, cimigenoside, and ginsenoside Rb1, respectively.

### 2.5. Cell Viability and Proliferation

XTT assays were used to determine the cytotoxicity of PSM on HaCaT cells. After treating the cells with emodin, genipin, chlorogenic, cimigenoside, ginsenoside Rb1, or PSM (10, 50 *µ*M) for 24 h, 50 *µ*l of an XTT solution (Sigma-Aldrich, St. Louis, MO) was added and incubated for 4 h. The absorbance was then measured at 450 nm (using a reference wavelength of 670 nm) using a microplate reader (Tecan, Männedorf, Switzerland). The proliferation of HaCaT cells was evaluated using a 5-bromo-2′-deoxyuridine (BrdU) proliferation assay kit (Cell Biolabs, San Diego, CA), which measured DNA synthesis. The HaCaT cells were pretreated with PSM (10, 50 *µ*M) or DEX (10 *μ*M) for 1 h and then stimulated with IL-22 (100 ng/ml) for 24 h. The cells were then incubated with 10 *µ*l of a BrdU-labeling solution for 4 h. The absorbance was measured at 450 nm using a microplate reader (Tecan, Männedorf, Switzerland).

### 2.6. Enzyme-Linked Immunosorbent Assay (ELISA)

Skin tissues were homogenized with ice-cold tissue extraction reagent, centrifuged at 10,000 ×g for 20 min, and the supernatants were collected. The levels of IFN-*γ*, IL-1*β*, and IL-6 in the skin tissues were measured using commercial ELISA kits according to the manufacturer's protocols. For the in vitro experiments, the levels of CXCL8, CXCL10, and CCL20 in the culture media from the HaCaT cells were evaluated using commercial kits according to the manufacturer's protocol. The absorbance was measured at 450–550 nm using an automated microplate reader (Tecan, Männedorf, Switzerland).

### 2.7. Cell Cycle Analysis

Cell cycle analyses were performed using a Muse Cell Cycle kit (Merck KGaA, Darmstadt, Germany) according to the manufacturer's protocol. The HaCaT cells were pretreated with PSM (50 *μ*M) or DEX (10 *μ*M) for 1 h and stimulated with IL-22 (100 ng/ml) for 48 h. After treatment, the cells were harvested and fixed with 1 ml of 70% cold ethanol at −20°C for 3 h, and then treated with 200 *μ*l of Muse Cell Cycle reagent and incubated for 30 min at room temperature in the dark. The percentage of cells in the G0/G1, S, and G2/M phases was then measured using a Muse Cell Analyzer (Merck KGaA, Darmstadt, Germany). The proliferation index (PI) was calculated using the formula: PI = (S + G2/M)/(G0/G1 + S + G2/M) [16].

### 2.8. Western Blot Analysis

HaCaT cells were lysed with a RIPA lysis buffer, containing protease and phosphatase inhibitors (Atto, Tokyo, Japan). After sonication, the cell lysates were centrifuged at 8000 x g for 10 min, and the supernatants were collected. The protein concentration was determined using a Bradford protein assay reagent (Bio-Rad, CA, USA). Subsequently, 30–50 *µ*g of the total proteins were separated by 5–10% SDS-PAGE electrophoresis and transferred to polyvinylidene difluoride membranes (Merck Millipore, Carrigtwohill, Ireland). After blocking for two hours in 5% skim milk in 1X PBS at room temperature, the membranes were incubated with the primary antibodies followed by the secondary antibody horseradish peroxidase-conjugated anti-IgG. All membranes were detected by enhanced chemiluminescence (Bio-Rad, CA, USA). The band intensities of the proteins were quantified using the GelPro V3.1 software (Media Cybernetics, MD, USA).

### 2.9. Statistical Analysis

All experiments were conducted in at least three independent experiments. The results are presented as the means ± standard deviation (SD) followed by the statistical significance (Student's *t*-test for unpaired experiments) with a *p* value <0.05.

## 3. Results

### 3.1. Effects of PSM on Psoriasis-Like Symptoms in IMQ-Induced C57BL/6 Mice

**Figure 2:**
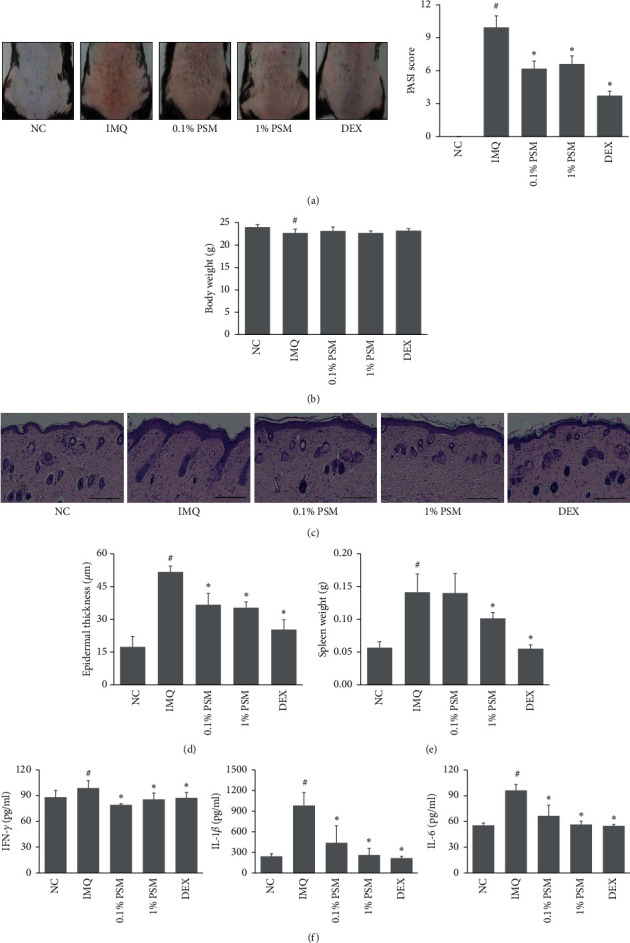
Effects of PSM on psoriasis-like symptoms in IMQ-induced C57BL/6 mice. (a) Representative images of the five groups. Scoring was performed using the Psoriasis Area Severity Index (PASI). (b) The body weights were measured. (c) Hematoxylin and eosin- (H&E-) stained sections of dorsal skin samples (magnification 100x, scale bar: 200 *μ*m). (d) Quantification of the epidermal thickness. (e) The spleen weights were measured. (f) Levels of IFN-*γ*, IL-1*β*, and IL-6 in the skin were determined using commercial ELISA kits. The results are presented as the means ± SDs (*n* = 7 per experiment). ^#^*P* < 0.05 vs. the NC group. ^*∗*^*P* < 0.05 vs. the IMQ group.

The effects of PSM on IMQ-induced psoriasis-like symptoms, redness, scaling, and thickness were assessed. At the end of the six-day treatment period, the dorsal skins of the IMQ-treated mice showed the typical features of psoriasis. The PASI scores were calculated based on measurements of scaling, redness, and thickness in the skin lesion. The PSM treatment attenuated the PASI score significantly compared to the IMQ-treated group (Figure 2(a)). The mice treated with PSM had significantly lower body weights compared to normal control (Figure 2(b)). H&E staining showed that PSM prevented IMQ-induced epidermal thickening (Figures 2(c) and 2(d)). The IMQ group showed a significant increase in spleen weight, which was reduced significantly by the PSM treatment (Figure 2(e)). To evaluate the effects of PSM on skin inflammation, ELISA was performed to measure the levels of cytokines in the skin lesions. These results showed that IMQ stimulation increased the expression of proinflammatory cytokines IFN-*γ*, IL-1*β*, and IL-6 in skin lesions. In contrast, treatment with PSM reduced the levels of these cytokines significantly (Figure 2(f)).

### 3.2. Effects of PSM on Proinflammatory Chemokine Production in M5-Stimulated HaCaT Cells

**Figure 3:**
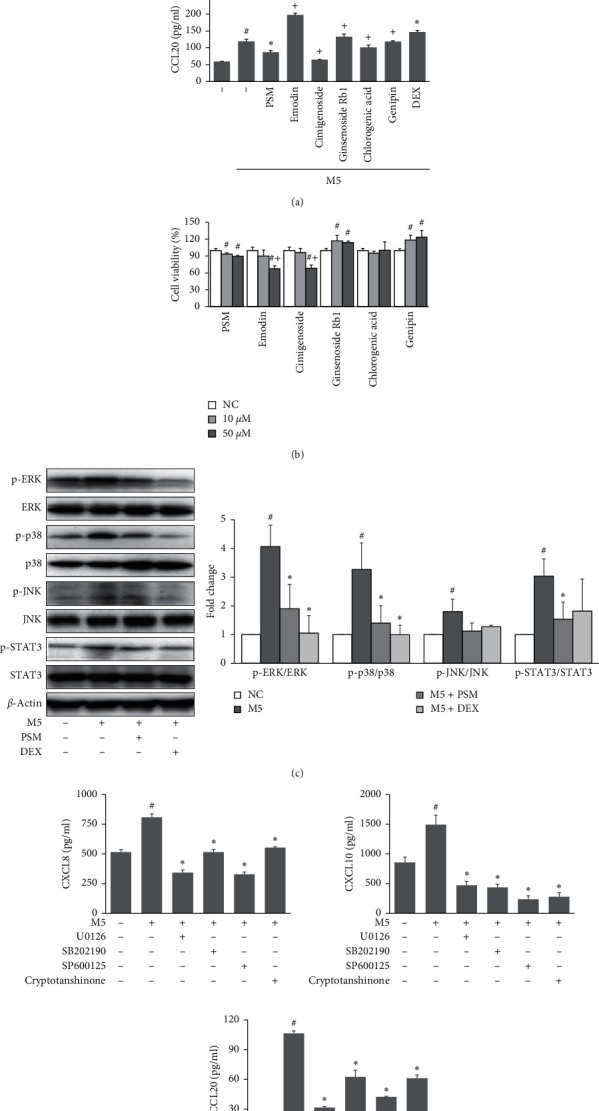
Effects of PSM on the proinflammatory chemokine expression and related signaling pathways in M5-stimulated HaCaT cells. (a) HaCaT cells were pretreated with emodin, genipin, chlorogenic, cimigenoside, ginsenoside Rb1 (10 *μ*M), PSM (50 *μ*M), or dexamethasone (DEX, 10 *μ*M) for 1 h and then stimulated with M5 (IL-1*α*, IL-17A, IL-22, oncostatin M, and TNF-*α*, 10 ng/ml each) for 24 h. The CXCL8, CXCL10, and CCL20 levels in the culture supernatants were determined using commercial ELISA kits. (b) The effects of PSM on cell viability was assessed using an XTT assay. HaCaT cells were treated with emodin, genipin, chlorogenic, cimigenoside, ginsenoside Rb1, or PSM (10, 50 *μ*M) for 24 h. (c) HaCaT cells were pretreated with PSM (50 *μ*M) or DEX (10 *μ*M) for 1 h and then stimulated with M5 for 30 min. Protein expression of p-ERK, p-p38, p-JNK, and p-STAT3 were assessed by Western blotting, and the band intensities were normalized versus ERK, p38, JNK, and STAT3, respectively. (d) HaCaT cells were pretreated with U0126, SB202190, SP600125, and cryptotanshinone (10 *μ*M) for 1 h and then stimulated with M5 for 24 h. The CXCL8, CXCL10, and CCL20 levels in the culture supernatants were determined using commercial ELISA kits. ^#^*P* < 0.05 vs. normal controls. ^*∗*^*P* < 0.05 vs. M5-treated cells. ^+^*P* < 0.05 vs. M5 + PSM-treated cells.

HaCaT cells were stimulated with a cocktail of five proinflammatory cytokines (M5), including IL-1*α*, IL-17A, IL-22, oncostatin M, and TNF-*α* that has been demonstrated as an in vitro model of psoriasis [17]. As shown in Figure 3(a), the preincubation of PSM inhibited the M5-induced upregulation of CXCL8, CXCL10, and CCL20 in HaCaT cells significantly, without affecting the cell viability (Figure 3(b)). Interestingly, PSM exhibited the synergistic effects of the five single compounds on inhibiting CXCL8 production and overcame the adverse effects of emodin and ginsenoside Rb1 on CCL20 production in M5-stimulated HaCaT cells (Figure 3(a)). Moreover, cytotoxicity was decreased when 50 *μ*M of PSM was treated compared with 50 *μ*M of emodin and cimigenoside (Figure 3(b)).

### 3.3. Effects of PSM on Activation of MAPK and STAT3 Signaling Pathways in M5-Stimulated HaCaT Cells

Previous studies suggested that the activation of MAPK and STAT3 signaling pathways promoted the development of psoriasis. [18, 19]. Therefore, this study examined the effects of PSM on the phosphorylation of MAPK and STAT3 in M5-treated HaCaT cells. As shown in Figure 3(c), pretreatment with PSM (50 *μ*M) decreased the M5-induced phosphorylation of ERK1/2, p38, and STAT3 significantly in HaCaT cells. U0126, SB202190, SP600125, and cryptotanshinone, which are inhibitors of ERK, p38, JNK, and STAT3, respectively, were used to confirm the role of the MAPK and STAT3 signaling pathways in M5-induced inflammatory chemokine production. As shown in Figure 3(d), treatment with these inhibitors suppressed the production of CXCL8, CXCL10, and CCL20 in M5-stimulated HaCaT cells.

### 3.4. Effects of PSM on Proliferation in IL-22-Stimulated HaCaT Cells

**Figure 4:**
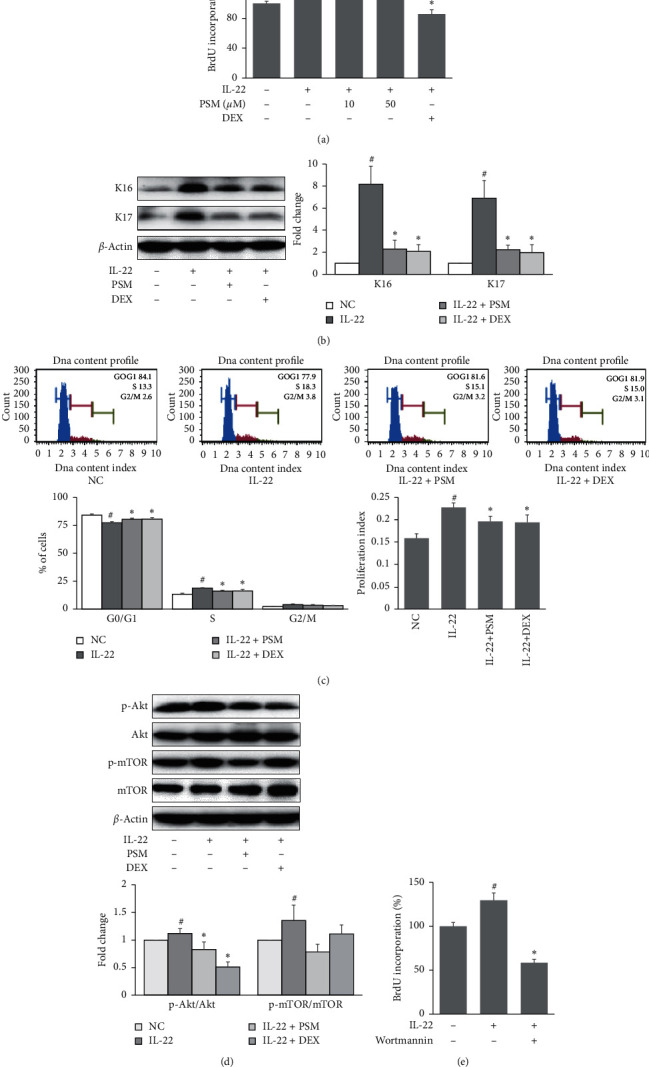
Effects of PSM on proliferation and the related signaling pathways in IL-22-stimulated HaCaT cells. (a) HaCaT cells were pretreated with PSM (10, 50 *μ*M) or DEX (10 *μ*M) for 1 h and then stimulated with IL-22 (100 ng/ml) for 48 h. Cell proliferation was measured using a BrdU proliferation assay kit. (b) Protein expression of K16 and K17 were assessed by Western blot analysis, and the band intensities were normalized versus *β*-actin. (c) The effects of PSM on the cell cycle were assessed using a Muse Cell Cycle kit. Proliferation index (PI) = (S + G2/M)/(G0/G1+S + G2/M). (d) HaCaT cells were pretreated with PSM (50 *μ*M) or DEX (10 *μ*M) for 1 h and then stimulated with IL-22 for 30 min. Protein expressions of p-Akt and p-mTOR were assessed by Western blot, and the band intensities were normalized versus Akt and mTOR, respectively. (e) HaCaT cells were pretreated with wortmannin (1 *μ*M) for 1 h and then stimulated with IL-22 (100 ng/ml) for 48 h. Cell proliferation was measured using a BrdU proliferation assay kit. The results are presented as the means ± SDs (*n* = 3 per experiment). ^#^*P* < 0.05 vs. normal controls. ^*∗*^*P* < 0.05 vs. IL-22-treated cells.

Hyperproliferation of keratinocytes is a hallmark of the pathogenesis of psoriasis [20]. This study examined the effects of PSM on IL-22-induced proliferation in HaCaT cells. The BrdU incorporation assay showed that pretreatment with PSM suppressed the proliferation induced by IL-22 significantly (Figure 4(a)). Expression of the proliferation markers, including K16 and K17, was also evaluated. Pretreatment with PSM decreased the IL-22-induced upregulation of K16 and K17 in HaCaT cells significantly (Figure 4(b)). Moreover, IL-22 increased the percentage of cells in the S phase and decreased the percentage of cells in the G0/G1 phase. In contrast, treatment with PSM had opposite effects (Figure 4(c)), indicating the antiproliferative effects of PSM on IL-22-stimulated HaCaT cells. The proliferation index (PI) was also calculated. A pretreatment with PSM reduced the IL-22-induced increase in PI significantly (Figure 4(c)).

### 3.5. Effects of PSM on Activation of Akt/mTOR Signaling Pathways in IL-22-Stimulated HaCaT Cells

Akt/mTOR is an important signaling pathway involved in keratinocyte proliferation [21]. This study examined the effects of PSM on the phosphorylation of Akt and mTOR. IL-22 increased the levels of phospho-Akt and phospho-mTOR significantly, whereas preincubation with PSM reduced the phosphorylation of Akt and mTOR markedly, as shown in Figure 4(d). Wortmannin is a prototypical inhibitor of PI3K, which also inhibits the phosphorylation of Akt and mTOR [22]. Wortmannin was used to confirm the role of Akt/mTOR signaling in IL-22-induced proliferation in HaCaT cells. As shown in Figure 4(e), pretreatment with wortmannin reduced the hyperproliferation of M5-stimulated HaCaT cells significantly.

## 4. Discussion

Psoriasis is a chronic inflammatory skin disease that produces physical, psychological, and economic burden to a patient throughout life. Topical therapy, systemic therapy, and biologics are used to treat psoriasis, but patient satisfaction is still low [23]. There is promising evidence that traditional multiherbal formulas are effective in treating and managing psoriasis [8]. The use of a multiherbal formula is expected to lead to complex interactions between multiple compounds and multiple targets, leading to better efficacy [9]. On the other hand, there are limitations to standardization and internationalization of the traditional multiherbal formula owing to the unclear component and underlying mechanism of action. Therefore, it is important to identify bioactive compounds that are effective in multiherbal formulas for developing quality control methods and uncovering therapeutic mechanisms [13, 24].

When using a traditional multiherbal formula to diagnose and treat psoriasis, rather than emphasizing disease classification, it is based on the patient's Zheng (syndrome type or patterns) [14, 25]. Herbal combinations of Rhei Radix et Rhizoma, Gardeniae Fructus, Cimicifugae Rhizoma, and Ginseng Radix are used to treat the symptoms of heat and dryness, the representative Zheng of psoriasis [13, 24]. Moreover, the main compounds from these medicinal herbs are known to have anti-inflammatory, antiproliferative, and/or antiatopic dermatitis activity [26–30]. Emodin is an anthraquinone derivative isolated from Rheum Rhizoma that has anti-inflammatory and antiproliferative effects in lung diseases [31, 32]. Genipin is an active compound in Gardeniae Fructus and was reported to suppress inflammation and oxidative stress in a dextran sulfate sodium-induced colitis mouse model [28]. Chlorogenic acid, another compound from Gardeniae Fructus, had anti-inflammatory activities on oxazolone-induced atopic dermatitis in mice [27]. Cimigenoside is a cimigenol derivative compound isolated from Cimicifugae Rhizoma that has antiproliferative activity [29]. Ginsenoside Rb1 is the main constituent of Ginseng Radix and has beneficial effects on skin damage in both in vivo and in vitro models [30]. Therefore, it was hypothesized that a combination of compounds derived from these herbs might be useful in the treatment of psoriasis.

In psoriasis, keratinocytes participate in cutaneous inflammatory responses by producing various chemokines that direct immune cells. CXCL8 and CXCL10 expressions have been reported to increase in the lesional skin from patients with psoriasis. These two chemokines are involved in the recruitment of neutrophils and Th1 cells to the inflammatory skin lesions [33]. CCL20 is strongly expressed in psoriatic keratinocytes and binds specifically to CCR6 receptors on Th17 cells to trigger the production of IL-17 and IL-22 from these cells [34]. In the present study, ginsenoside Rb1 inhibited CXCL8 and CXCL10 slightly but did not have any inhibitory effect on CCL20. A previous study from other researchers also reported that ginsenoside Rb1 did not significantly show the antipsoriatic activity [35]. Emodin inhibited CXCL8 and CXCL10 but rather increased CCL20 production. Nevertheless, the natural compound mixture, PSM, inhibited the production of CXCL8 more effectively than every single compound. PSM significantly inhibited the production of CXCL10 and CCL20 in M5-stimulated HaCaT cells. As with previous research results, the current results showed that a combination of compounds could have synergistic effects and act on multitargets [36].

Herbal therapy can benefit the treatment of skin diseases, but toxicity remains an important concern [37]. Emodin is an anthraquinone derivative with several biological activities but can cause liver toxicity and induce apoptosis of human T cells [38, 39]. The results of the present study indicated that a combination of compounds could reduce toxicity and side effects. Cytotoxicity was observed when treated with 50 *μ*M of emodin and cimigenoside (<60%), but reduced cytotoxicity of PSM was observed. This may be related, at least partially, to the cytoprotective efficacy of ginsenoside Rb1 and genipin. Moreover, in the PSM-treated mice, weight loss, deterioration of skin symptoms, and mortality did not increase. Although further research is needed to confirm the safety of PSM in the treatment of psoriasis, topical application of PSM in this study appears to be safety in the concentration range of 0.1%–1.0%.

As the common doses of commercially available topical corticosteroids for the treatment of psoriasis ranged from 0.05%–1%, PSM 0.1% and 1% were chosen for the topical application [6]. The topical application of PSM to C57BL/6 mice treated with IMQ suppressed the psoriasis-like symptoms in terms of redness, scaling, and skin thickening. The inflammatory infiltrates composed of dendritic cells (DC), T cells, macrophages, and neutrophils are prominent in psoriatic lesions. These immune cells produce a variety of proinflammatory cytokines, such as IFN-*γ*, IL-1*β*, IL-6, IL-17, and IL-22 [1]. IFN-*γ* is a notable cytokine in psoriatic lesions that can induce the upregulation of Th1 and dendritic cells chemokines, such as CXCL10 and CCL2, which are expressed prominently in psoriasis [40]. IL-1*β* and IL-6 are other highly expressed cytokines in the psoriatic lesional skin, which are produced not only by immune cells but also by keratinocytes [41]. IL-1*β*, together with IL-23, is involved in Th17 differentiation and the production of IL-17 and IL-22, which are crucial in the pathogenesis of psoriasis [42]. IL-6 is required for IL-22-mediated skin inflammation and epidermal hyperplasia in psoriasis by regulating the expression of IL-22 receptor chain IL-22R1 [43]. The inhibition of IL-6 signaling by tofacitinib showed a significant improvement in patients with moderate-to-severe psoriatic symptoms [44]. The levels of these cytokines in skin lesions were measured. The results showed that PSM reduced the protein levels of IFN-*γ*, IL-1*β*, and IL-6 significantly. These results suggest that a PSM treatment ameliorated erythematous scaly plaque by suppressing the inflammatory cytokines.

The effects of PSM on the STAT3 and MAPK (ERK1/2, p38, JNK) signaling pathways were examined to clarify the molecular mechanism underlying the anti-inflammatory effects of PSM. STAT3 is crucial for the induction of inflammation and keratinocyte proliferation in psoriatic skin lesions; thus, STAT3 is considered a potent target for psoriasis therapy [19]. Inhibiting the activation of STAT3 using a topical inhibitor affected not only the animal model of psoriasis but also patients with actual psoriasis [19]. The levels of the phosphorylated forms of ERK1/2 and p38, not JNK, were also significantly higher in the lesional psoriatic skin [45]. The suppression of MAPK signaling pathways by a natural compound, cimifugin, significantly attenuated inflammation in both in vitro and in vivo models of psoriasis [46]. In the present study, PSM blocked the phosphorylation of ERK1/2, p38, and STAT3 significantly in M5-stimulated HaCaT cells, suggesting that the therapeutic action of PSM might be through inhibition of the ERK1/2, p38, and STAT3 signaling pathways.

IL-22 can induce hyperproliferation, cell migration, and disrupt the terminal differentiation of keratinocytes, resulting in pathological epidermal hyperplasia in psoriasis. IL-22 expression is upregulated in the skin lesions and serum of patients with psoriasis, and it is strongly related to the disease severity [47]. IL-22-induced keratinocyte hyperproliferation is regulated by the PI3K/Akt/mTOR signaling pathway, and targeting this pathway is a potent approach for psoriasis treatment [21, 48]. Histologically, PSM treatment ameliorated epidermal hyperplasia significantly in IMQ-induced C57BL/6 mice. A PSM treatment reduced the expression of K16 and K17 and hyperproliferation markers by inhibiting the phosphorylation of Akt and mTOR in IL-22-simulated HaCaT cells. Moreover, PSM inhibited the S phase of the cell cycle in IL-22-stimulated HaCaT cells. As DNA replication in S phase is a key event of cell proliferation, PSM may partially prevent cells from entering the S phase, thereby decreasing keratinocyte proliferation [49].

The present study has some limitations. Further studies will be needed to determine the ideal topical vehicle to increase the bioavailability of PSM. The traditional herbal formula, antelope horn powder, contains the same amount of Rhei Radix et Rhizoma, Gardeniae Fructus, Cimicifugae Rhizoma, and Ginseng Radix. Hence, active compounds isolated from each herb were mixed in equal amounts in PSM. Further studies will be needed to determine the appropriate concentration ratio between each compound.

## 5. Conclusion

These results showed that the topical application of PSM alleviates psoriasis-like symptoms in IMQ-induced C57BL/6 mice, with no apparent side effects. Pretreatment with PSM inhibited the M5-induced production of proinflammatory chemokines by inhibiting ERK1/2, p38, and STAT3 signaling pathways in HaCaT cells. The PSM pretreatment also suppressed proliferation by blocking the Akt/mTOR signaling pathway in IL-22-stimulated HaCaT cells. These results suggest that PSM is a potential candidate for the treatment of psoriasis.

## Data Availability

The data used to support this study are included within the article.

## Abbreviations

IMQ: Imiquimod
CXCL8: C-X-C motif chemokine ligand 8
CCL20: C-C motif chemokine ligand 20
DEX: Dexamethasone
ELISA: Enzyme-linked immunosorbent assay.
